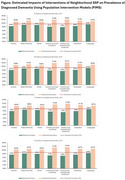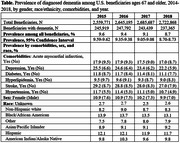# Neighborhood Spatial Social Polarization and Dementia Disparities Among U.S. Medicare Beneficiaries

**DOI:** 10.1002/alz70861_108433

**Published:** 2025-12-23

**Authors:** Hoda S Abdel Magid, Mengya Xu

**Affiliations:** ^1^ University of Southern California, Los Angeles, CA USA

## Abstract

**Background:**

Alzheimer’s Disease and related dementias (ADRD) disproportionately affect racial and ethnically minoritized populations. Spatial Social Polarization (SSP)—measured via the Index of Concentration at the Extremes (ICE)—captures neighborhood‐level privilege and deprivation across domains like race, income, and education. SSP is a flexible and predictive neighborhood‐level measure of health disparities compared to indices like the Gini coefficient. While SSP has been linked to other chronic conditions, its association with ADRD remains understudied.

**Method:**

We analyzed a 20% sample of Medicare beneficiaries enrolled in Traditional Medicare (Parts A, B, and D) from 2014 to 2019, identifying those aged 67+ with continuous enrollment. Dementia diagnosis was determined using a combination of diagnosis codes for dementia‐related conditions and prescription drug records. The final analytic sample included 2.5–2.7 million beneficiaries per year from 2015‐2018, with an average age of 76.

SSP was measured across domains of race, income, education, language, and homeownership at the ZIP code level. Descriptive analyses examined disparities in dementia prevalence across sociodemographic groups. Mixed‐effects regression models assessed the association between neighborhood SSP and dementia prevalence, adjusting for age, race, sex, and comorbidities. We applied population intervention models (PIMs) using the parametric g‐formula to estimate the potential population‐level impact of eliminating SSP disparities.

**Result:**

Among 2.5–2.7 million beneficiaries annually, dementia prevalence declined from 9.6% in 2015 to 8.7% in 2018, despite growth in the beneficiary population. ADRD prevalence remained higher among Black and Hispanic individuals, women, and those with comorbidities.

Greater neighborhood privilege (higher ICE scores) was consistently associated with lower dementia prevalence (β ∼ ‐0.04 to ‐0.06; *p* < 0.01). PIM simulations suggested that reducing deprivation in ICE domains could lower dementia prevalence by up to 1.9%. Conversely, hypothetical increases in neighborhood deprivation could raise prevalence by as much as 2.8%. For example, moving all residents to predominantly Black neighborhoods increased predicted prevalence by 1.8–2.0%, while moving them to predominantly White neighborhoods decreased it by 0.5–0.6%.

**Conclusion:**

Neighborhood‐level deprivation and racial segregation are associated with increased ADRD prevalence, independent of individual characteristics. SSP represents a modifiable, policy‐relevant exposure that may contribute to persistent dementia disparities.